# Habitual Nightly Fasting Duration, Eating Timing, and Eating Frequency are Associated with Cardiometabolic Risk in Women

**DOI:** 10.3390/nu12103043

**Published:** 2020-10-04

**Authors:** Nour Makarem, Dorothy D. Sears, Marie-Pierre St-Onge, Faris M. Zuraikat, Linda C. Gallo, Gregory A. Talavera, Sheila F. Castaneda, Yue Lai, Junhui Mi, Brooke Aggarwal

**Affiliations:** 1 Department of Medicine, Columbia University Irving Medical Center, New York, NY 10032, USA; ms2554@cumc.columbia.edu (M.-P.S.-O.); fmz2105@cumc.columbia.edu (F.M.Z.); baf2108@cumc.columbia.edu (B.A.); 2 Sleep Center of Excellence, Columbia University Irving Medical Center, New York, NY 10032, USA; 3 College of Health Solutions, Arizona State University, Tempe, AZ 85004, USA; dorothy.sears@asu.edu; 4 Department of Medicine, UC San Diego San Diego School of Medicine, La Jolla, CA 92093, USA; 5 Department of Family Medicine and Public Health, UC San Diego School of Medicine, La Jolla, CA 92093, USA; 6 Moores Cancer Center, UC San Diego, La Jolla, CA 92093, USA; 7 Department of Psychology, San Diego State University, San Diego, CA 92182, USA; lgallo@sdsu.edu (L.C.G.); GTALAVERA@sdsu.edu (G.A.T.); scastaneda@sdsu.edu (S.F.C.); 8 Department of Biostatistics, Mailman School of Public Health, Columbia University Irving Medical Center, New York, NY 10032, USA; yl4359@cumc.columbia.edu (Y.L.); jm4998@cumc.columbia.edu (J.M.)

**Keywords:** nightly fasting duration, circadian, eating timing, eating frequency, cardiometabolic risk, diabetes, cancer, cardiovascular health, women

## Abstract

Nightly fasting duration (NFD) and eating timing and frequency may influence cardiometabolic health via their impact on circadian rhythms, which are entrained by food intake, but observational studies are limited. This 1-year prospective study of 116 US women (33 ± 12y, 45% Hispanic) investigated associations of habitual NFD and eating timing and frequency with cardiovascular health (CVH; American Heart Association Life’s Simple 7 score) and cardiometabolic risk factors. NFD, eating timing and frequency, and nighttime eating levels were evaluated from 1-week electronic food records completed at baseline and 1 y. In multivariable-adjusted linear regression models, longer NFD was associated with poorer CVH (β = −0.22, *p* = 0.016 and β = −0.22, *p* = 0.050) and higher diastolic blood pressure (DBP) (β = 1.08, *p* < 0.01 and β = 1.74, *p* < 0.01) in cross-sectional and prospective analyses, respectively. Later timing of the first eating occasion at baseline was associated with poorer CVH (β = −0.20, *p* = 0.013) and higher DBP (β = 1.18, *p* < 0.01) and fasting glucose (β = 1.43, *p* = 0.045) at 1 y. After adjustment for baseline outcomes, longer NFD and later eating times were also associated with higher waist circumference (β = 0.35, *p* = 0.021 and β = 0.27, *p* < 0.01, respectively). Eating frequency was inversely related to DBP in cross-sectional (β = −1.94, *p* = 0.033) and prospective analyses (β = −3.37, *p* < 0.01). In cross-sectional analyses of baseline data and prospective analyses, a higher percentage of daily calories consumed at the largest evening meal was associated with higher DBP (β = 1.69, *p* = 0.046 and β = 2.32, *p* = 0.029, respectively). Findings suggest that frequent and earlier eating may lower cardiometabolic risk, while longer NFD may have adverse effects. Results warrant confirmation in larger multi-ethnic cohort studies with longer follow-up periods.

## 1. Introduction

Mounting evidence suggests that eating and fasting patterns influence cardiometabolic heath [1,2]. Compelling data from animal models and short-term clinical trials in humans indicate that time-restricted feeding (TRF), the practice of eating during a restricted window of time and fasting thereafter, has favorable effects on body weight, glucose metabolism, and blood pressure (BP), independent of energy intake [2,3,4]. The circadian timing of food intake is also related to cardiometabolic risk; particularly, an earlier eating period and less nighttime eating has been linked to better cardiometabolic health, since fasting/eating cycles that are chronically misaligned with 24-h light/dark cycles can lead to disrupted circadian rhythms, a risk factor for obesity, type 2 diabetes, hypertension, and cardiovascular disease (CVD) [1,2]. Beyond the timing of food intake, the frequency of eating episodes has been proposed to play a role in cardiometabolic health, with some evidence of beneficial effects of frequent meals on weight-related outcomes [5]; however, mixed results preclude a scientific consensus regarding the role of eating frequency in obesity risk [1].

Although there is promising emerging evidence that dietary patterns that are intentionally mindful of the daily span of eating/fasting duration and the timing and frequency of eating may represent a modifiable lifestyle approach for the management of cardiometabolic risk factors [1], a number of scientific gaps remain. First, the protective effects of TRF on cardiometabolic health are primarily demonstrated in clinical investigations with limited sample sizes and short follow-up (typically 8–12 weeks and one for 16 weeks), and many are conducted in “high-risk” populations such as adults with overweight, obesity, or prediabetes, some of which do not include women [2,4,6,7,8]. Secondly, observational data, particularly among ethnically diverse US adults, are limited [9,10] and only investigated outcomes such as body mass index (BMI), hemoglobin A1c (HbA1c), C-reactive protein, and breast cancer recurrence [1,11]. Evidence is non-existent for associations with overall cardiovascular health (CVH), a strong predictor of future CVD risk [12], and other clinically relevant cardiometabolic risk factors, such as central adiposity, BP, fasting glucose, and blood lipids.

The majority of previous observational studies in this research area are cross-sectional and rely on dietary data from a limited number of 24-h recalls [1,13], assuming that these aspects of diet are stable over time and that one or few days of diet data are representative of habitual fasting and eating patterns. In addition, existing observational studies of eating frequency have produced conflicting results for obesity risk, though protective associations have generally been documented for other cardiometabolic risk factors, particularly glycemic indicators [1]. Several of these research gaps were acknowledged in a 2017 American Heart Association (AHA) scientific statement addressing the role of meal timing and frequency in cardiometabolic health [1], highlighting the need for additional research on this topic, particularly in racially/ethnically diverse samples. Thus, the purpose of this observational study was to investigate cross-sectional and prospective associations of habitual nightly fasting duration (NFD), timing of the first and last eating occasions, and eating frequency with overall CVH and clinically relevant cardiometabolic risk factors in a community-based sample of US women.

## 2. Materials and Methods

### 2.1. Design and Study Population

Participants for this analysis were a sub-set of women from the AHA Go Red for Women Strategically Focused Research Network (SFRN) at Columbia University Irving Medical Center (CUIMC). The AHA Go Red for Women SFRN, which has been described in detail elsewhere [14,15], is a 1-year prospective cohort study of English- and Spanish-speaking non-pregnant women, aged 20–76 y, that was designed to investigate the associations of psychosocial factors with sleep patterns and cardiometabolic risk. Women from that cohort were recruited to participate in this ancillary study, designed to gain additional insight into the role of eating patterns in cardiometabolic risk.

Overall, 116 women (45% Hispanic), aged 20–64 y, consented to participate in this ancillary study, which required the completion of a 1-week electronic food record and provision of a blood sample to measure HbA1c at baseline and 1-y follow-up, in addition to the measures collected as part of the main study. All research activities were approved by the CUIMC Institutional Review Board (IRB; protocol number AAAQ8196), and conducted in accordance with the Declaration of Helsinki. Participants provided written informed consent prior to participating in the study and were compensated following completion of both the main and ancillary studies. 

### 2.2. Habitual Nightly Fasting Duration and Eating Timing and Frequency

Women completed a 7-day electronic food record using the Automated Self-Administered 24-h (ASA24) Dietary Assessment Tool, a free, web-based tool developed by the National Institutes of Health that enables automatically coded multiple day food diaries [16]. ASA24 is modeled upon the USDA Automated Multiple-Pass Method and generates detailed individual-level diet data files with information on diet quantity and quality as well as timing, frequency, and location of eating occasions. This tool generates high-quality dietary intake data in a range of settings and has demonstrated acceptable validity and reliability [16].

In this study, participants were asked to record intake on seven consecutive days to capture weekday–weekend differences in diet and better estimate habitual fasting and eating patterns. Email reminders from study staff were sent throughout the week to maximize data completeness. Overall, 91% and 96% of participants provided at least 6 days of diet data at baseline and 1-y follow-up, respectively. Time-stamped diet data were used to extract the daily times of the first and last eating occasions, defined as consuming ≥ 25 kcal, as reported previously [9,11]. This information was used to calculate the eating duration for each day. NFD was subsequently computed, as 24 h minus the eating duration for each day [10,11], and the average NFD across all days was calculated. The number of eating occasions on each reported day was used to estimate average eating frequency. To capture the association between level of nighttime eating and cardiometabolic risk, we extracted the percentage of daily calories consumed at the largest evening meal, which was identified by participants as “dinner” and/or “supper” in their food record.

### 2.3. Cardiovascular Health and Cardiometabolic Risk Factors

Participants attended a clinic visit where anthropometric measurements were obtained. Height and weight were used to compute BMI (kg/m^2^). Waist circumference (WC) (inches) was measured just above the iliac crest using a standard measuring tape. Fasting blood samples were collected and sent to the Biomarker Core Laboratory at CUIMC to assess fasting glucose and blood lipids (total, low-density lipoprotein (LDL), and high-density lipoprotein (HDL) cholesterol and triglyceride levels) using standardized procedures. HbA1c was measured in whole blood using a DCA vantage analyzer (Siemens Diagnostics STARTA1C DCA Vantage^®^ Analyzer). Resting systolic blood pressure (SBP) and diastolic blood pressure (DBP) were measured in duplicate using a hospital-grade automated BP monitor (Omron 5 Series Upper Arm [BP742]) while participants were sitting, legs uncrossed, and relaxed for at least 5 min.

Participants also provided information on lifestyle behavior history using validated questionnaires. Smoking status (current vs. former/never smoker) was reported on a standardized health questionnaire. Habitual dietary intake over the past year was measured using the Block Brief Food Frequency Questionnaire [17]. Women reported the average time, in hours/day, spent engaging in moderate to vigorous intensity physical activity on the International Physical Activity Questionnaire [18]. These data were used to compute the AHA Life’s Simple 7 (LS7) score, as a measure of overall CVH [19]. Briefly, women received a score of 2, 1, or 0 if their level of meeting the guideline for BMI, fasting glucose, blood cholesterol, BP, smoking, diet, and physical activity was high, moderate, or low, respectively [14,19]. Then, the seven component scores were summed to create the global LS7 score (range: 0–14), as previously published in this cohort [14,15], with higher scores reflecting more favorable CVH.

### 2.4. Socio-Demographic Variables

Age (y), race (White, Black, or Asian), ethnicity (Hispanic vs. non-Hispanic), education level (≤college degree vs. >college degree), and health insurance status were self-reported on a standard health questionnaire. 

### 2.5. Statistical Analysis

Descriptive statistics were generated for socio-demographic, dietary, and cardiometabolic factors at the baseline and 1-y assessments and were presented as mean ± SD for continuous variables and as % of the total sample for categorical variables. T-tests and Chi-square tests were used to examine differences in these participant characteristics at baseline vs. 1-y follow-up. Linear regression models adjusted for age, race/ethnicity, health insurance (as a measure of socio-economic status in this cohort), and physical activity were used to evaluate baseline and 1-y cross-sectional associations of NFD (per 1 h increase), eating frequency (per each additional eating occasion), clock time of the first and last eating occasions (per 30-min delay), and percentage of daily calories consumed at the largest evening meal (per 10% increase) with the LS7 score and cardiometabolic risk factors, assessed on the continuous scale. Prospective analyses, with adjustment for age, race/ethnicity, health insurance, and physical activity, were also conducted to evaluate whether baseline exposures predicted 1 y outcomes. Models with BP, glycemic indicators, and blood lipids as outcomes were additionally adjusted for BMI. Linear regression models to evaluate prospective associations of baseline exposures with outcomes at 1 y were additionally adjusted for baseline outcomes. SAS version 9.4 (Cary, NC, USA) was used for all analyses. A *p*-value < 0.05 was considered significant.

## 3. Results

### 3.1. Socio-Demographic and Clinical Characteristic of the Study Population

At baseline, the average age of participants was 33 ± 12 y (Table 1). More than three-quarters of the women reported belonging to a racial and/or ethnic minority group, and 45% reported Hispanic ethnicity. About two-thirds of the sample had an education level equivalent to or less than college, and 58% had health insurance. Prevalence of smoking was low in this cohort (< 7%), and women reported an average of 4.1 and 5.7 h/day of moderate-to-vigorous intensity physical activity at the baseline and 1-y assessments, respectively. The average AHA LS7 score corresponded to moderate CVH. In general, approximately half of the women had a BMI in the overweight or obese category, and 45% had an at-risk WC > 35 inches. At baseline, mean NFD was 12.4 ± 2.2 h, and the average time of the first and last eating occasions was 8:32 ± 1:34 h and 20:08 ± 1:45 h, respectively. Baseline eating frequency was 3.9 ± 1.1 eating occasions/day. Average time of first eating occasion was later, mean NFD was longer, and eating frequency was lower at 1 y vs. baseline (*p* < 0.01); whereas average time of last eating occasion was only slightly earlier at 1 y compared to baseline (*p* = 0.046).

### 3.2. Associations of Habitual Nightly Fasting Duration with Cardiovascular Health and Cardiometabolic Risk Factors

Results from linear regression models adjusted for age, race/ethnicity, health insurance, and physical activity using cross-sectional data from baseline and 1 y and prospective data to evaluate baseline NFD in relation to outcomes at 1-y are shown in Table 2. In cross-sectional analysis of 1 y data, each 1-h increase in NFD was related to higher SBP (β = 1.00, *p* = 0.055) and DBP (β = 1.08, *p* = 0.009) and to poorer CVH (β = −0.22, *p* = 0.016). Findings from prospective analyses were similar, as longer baseline NFD was associated with poorer CVH (β = −0.22, *p* = 0.050) and with 1.74 mmHg higher DBP (*p* = 0.007) at 1 y. Additional adjustment for BMI did not significantly alter these findings. After additionally adjusting prospective models for baseline levels of cardiometabolic outcomes, associations persisted and longer NFD was also related to higher WC (β = 0.35, *p* = 0.021).

### 3.3. Associations of Eating Timing with Cardiovascular Health and Cardiometabolic Risk Factors

The clock times of the first and last eating occasion that were used to estimate habitual NFD were examined in relation to the study outcomes. Cross-sectional analyses at the baseline and 1 y time points and prospective analyses for every 30-min delay in timing of the first eating occasion are shown in Table 3. In the cross-sectional analysis of baseline data, 30-min later timing of the first eating occasion was associated with 2.32 mg/dL higher triglyceride levels (*p* = 0.045). In cross-sectional analyses of 1 y data and prospective analyses, every 30-min delay in timing of the first eating occasion was associated with 0.53 mmHg (*p* = 0.042) and 1.18 mmHg higher DBP (*p* < 0.01), respectively. Similarly, a later eating start time was associated with poorer CVH in prospective analyses (β = −0.20, *p* = 0.013), and a borderline significant association was observed in the cross-sectional analysis of 1 y data (β = −0.11, *p* = 0.058). In addition, a 30-min delay in timing of the first eating occasion at baseline was associated with 1.43 mg/dl higher fasting glucose at 1 y (*p* = 0.045). Additional adjustment for BMI did not alter the statistical significance of most of these results, but cross-sectional associations with DBP at 1 y were attenuated. Moreover, in models that were further adjusted for baseline levels of cardiometabolic risk factors, each 30-min delay in timing of the first eating occasion was additionally associated with 0.27 inches higher WC (*p* = 0.009).

Although the timing of the last eating occasion was not significantly associated with the outcomes (data not shown), in cross-sectional analyses of baseline data and prospective analyses, a higher percentage of daily calories consumed at dinner and/or supper was associated with higher DBP (β = 1.69, *p* = 0.046 and β = 2.32, *p* = 0.029, respectively).

### 3.4. Associations of Eating Frequency with Cardiovascular Health and Cardiometabolic Risk Factors

Cross-sectional associations of eating frequency with study outcomes at baseline and 1 y and prospective associations of baseline eating frequency with 1-y outcomes are shown in Table 4. In cross-sectional analyses of baseline data and prospective analyses, each additional eating episode was associated with 1.94 mmHg (*p* = 0.033) and 3.37 mmHg lower DBP (*p* = 0.001), respectively. Associations between eating frequency and DBP remained significant even after adjustment for BMI and even after adjustment for baseline DBP levels in prospective models (β(SE) = −1.94, *p* = 0.020).

## 4. Discussion

In this community-based cohort of 116 relatively healthy US women, habitual NFD and the timing and frequency of eating occasions were related to overall CVH and cardiometabolic risk factors. Longer NFD was associated with poorer CVH and higher BP. Later clock time of the first eating occasion was related to poorer CVH and higher DBP and fasting glucose. A higher percentage of daily calories consumed at dinner and/or supper was related to higher DBP. Greater frequency of eating occasions was inversely associated with DBP. Notably, associations with BP demonstrated strong effect sizes in all analyses, which may have significant public health implications, given that high BP is the leading cause of CVD morbidity and mortality in the US [20]. Collectively, these data suggest that aspects of eating patterns, beyond the traditional diet quantity and quality metrics, may be of significance for lowering the risk of cardiometabolic disease in US women.

We demonstrate unfavorable associations between longer NFD and cardiometabolic risk, which are inconsistent with findings from clinical trials on TRF [2,8]. However, much of the experimental evidence demonstrating protective effects on body weight, glycemic regulation, and blood lipids is derived from male rodent models [3,21] or human clinical trials [4,6,7,22,23,24,25] with small sample sizes (n = 8 to 29). Most of these trials have been conducted in high-risk populations (e.g., adults with overweight, obesity, and prediabetes), and over-represented by men overall, who may have different metabolic risk profiles and lifestyle habits compared to the general population. In addition, the mean age of the populations in these trials tends to be older (>50 y). Contrary to findings from clinical trials, limited epidemiologic data on NFD and cardiometabolic risk have yielded inconsistent results [10,11,13,26,27]. A cross-sectional study among women participating in the 2009–2010 National Health and Nutrition Examination Survey (NHANES; n = 2212; mean age: 47 y) showed that every 3-h increase in NFD was associated with 20% lower odds of elevated HbA1c [10]. Similar to the NHANES cohort, each 2-h increase in NFD was associated with lower HbA1c levels in a prospective study of 2413 breast cancer survivors (mean age: 52 y) [11]. In that study, women with NFD < 13 h had lower mean BMI, though BMI results were not significant in adjusted linear models. However, a prospective analysis of the Adventist Health Study 2 (N = 50,660, mean age: 58 y), of which > 50% of men and women were vegetarian, showed that participants with NFD ≥18 vs. 12-17 h had a decrease in BMI over a mean follow up of 7.4 y [27].

Observational studies in Asian populations have yielded results conflicting with those conducted in the US, where Asian individuals are relatively under-represented [13,26]. In 1054 Japanese non−shift workers (mean age: 46 y), NFD was not associated with increased odds of having metabolic syndrome [26]. However, longer NFD (16 vs. 10–12 h) was associated with elevated cardiometabolic risk in the 2013–2017 Korean NHANES (mean age: 41 y) [13]. Distinct results across these different study populations suggest that associations of NFD with cardiometabolic risk may vary among racial and ethnic groups. Indeed, in exploratory analyses (data not shown), we found that associations of longer NFD with adverse cardiometabolic risk profiles are only significant in Hispanic women. One explanation could be the higher mean NFD among Hispanic vs. non-Hispanic women at baseline and 1 y (~1 h difference). More Hispanic women completed the 1-y follow-up assessment, which could also explain the stronger associations between NFD and cardiometabolic risk observed at 1 y, given the resulting slight increase in representation of Hispanic women at 1 y vs. baseline. Further, it is possible that NFD in an observational setting is a marker of another unassessed risk factor for cardiometabolic disease such as food insecurity. NHANES data demonstrate that 22% of Hispanic households vs. 14% of all US households are food insecure [28], and food insecurity has in turn been associated with elevated cardiometabolic risk in this population [29,30].

Another possible explanation for the discrepancies in study findings is that the association between NFD and cardiometabolic risk factors could be non-linear. In clinical trials, TRF regimens typically incorporate a fasting period of ≥14 h [4,8,25], and in the Adventist Health Study 2, a reduction in BMI was demonstrated in those with very high NFD (≥18 h), much higher than the average population [10,11]. It is possible that there is a threshold beyond which a longer NFD is beneficial or that a U—or J—shaped association exists that is not adequately captured in cohorts, similar to our sample herein, with a narrow range reported NFD and thus insufficient variability to test for such an association. Moreover, there may be other factors at play, including circadian timing of food intake and sleep duration. For example, data from the Korean NHANES showed that women who had longer NFD (≥12 h) and slept for <6 h had higher cardiometabolic risk, compared to women who had shorter NFD (<12 h) and sleep duration of 7–8 h, suggesting that sleep duration may be an effect modifier for associations of NFD with cardiometabolic risk [13]. Further, early TRF appears to be more protective than delayed TRF [7,31]. Indeed, we demonstrate in the present study that earlier timing of the first eating occasion may play a protective role in cardiometabolic health. This is consistent with the literature demonstrating that morning eating between 5 AM and 9 AM and not skipping breakfast are associated lower risk of overweight and obesity, abdominal obesity, glycemic dysregulation, metabolic syndrome, type 2 diabetes, and hypertension [1,13,27].

Nighttime eating, on the other hand, is hypothesized to elevate cardiometabolic risk by leading to disturbed circadian rhythms [1,2]. However, observational data on the timing and amount of food consumed in the evening in relation to cardiometabolic risk are limited and inconsistent [32]. These relations may also vary by sex, as night eating was associated with a 48% higher prevalence of metabolic syndrome in men but not women in the Korean NHANES [13]. Although timing of the last eating occasion was not related to any of the outcomes in this study, we uniquely demonstrate that a higher percentage of daily calories consumed at the self-defined evening meal was associated with higher DBP. Further, exploratory analyses revealed that a 10% increase in the proportion of daily calories consumed after 5PM and 8PM from baseline to 1 y was associated with an increase in BMI (β = 0.30, *p* = 0.050) and with an increase in both BMI (β = 0.34, *p* = 0.021) and WC (β = 0.48, *p* = 0.030), respectively. These results are consistent with previous observational data demonstrating a positive association between evening or dinner caloric intake and risk for obesity and metabolic syndrome [33,34,35], including in younger women [36]. In fact, a meta-analysis of observational studies reported a non-significant trend between BMI and evening intake [32]. However, it is possible that we underestimated the association of nighttime eating with cardiometabolic risk due to our sample size.

Our finding that frequent eating episodes are associated with lower BP is consistent with previous studies demonstrating an association between meal frequency and more favorable cardiometabolic health [1]. In cross-sectional studies of European, Asian, and US adults [11,37,38], frequent eating episodes were associated with lower odds of obesity, abdominal adiposity, and having high BP [38]. In prospective studies, consuming 1–2 vs. 3 meals/day predicted 26% higher risk of type 2 diabetes among men [39], and a higher number of eating episodes predicted lower BMI in women [11]. In our sample of women, a greater number of eating episodes was strongly related to lower DBP in the overall sample. In exploratory analyses (data not shown), we also detected significant inverse associations for meal frequency with BMI, WC, SBP, and DBP in Hispanic women only (p-interaction < 0.1), suggesting that associations may vary by ethnicity. This warrants further investigation in well-powered racially and ethnically diverse population-based cohort studies.

Strengths of our study include the prospective design, which enabled the investigation of cross-sectional and prospective associations in US women. The use of a 7-day electronic food record at baseline and 1 y, where women could enter information about their eating habits in real-time with visual aids for increased accuracy, is a major strength of this study. Given that prior observational studies have relied on diet data from one or few 24-h recalls [10,11,13], the collection of seven consecutive days of diet data at each time point was likely more representative of habitual intake in this study and enabled more accurate estimation of the exposures of interest. However, our study has some limitations that must be acknowledged. The 1-y follow-up duration may not be sufficient to capture significant changes in eating patterns and their association with changes in metabolic health across adulthood. Our sample size was modest, so it is possible that some findings may be due to chance. We had limited power to investigate differences in these associations by life stage, race, and ethnicity. Further, the possibility of residual confounding cannot be ruled out. For instance, seasonality may influence sleep timing patterns and subsequently eating timing patterns; the influence of seasonality and other circadian-related factors on these relations warrants further investigation. Finally, we recruited a community-based sample of generally younger women living in New York City, who may not be representative of the broader US population.

## 5. Conclusions

Eating patterns characterized by a longer NFD, later start time of the eating period, less frequent eating episodes, and a higher percentage of daily calories consumed in the evening may have adverse cardiometabolic consequences in women. These findings add to the inconsistent evidence base on NFD in relation to cardiometabolic risk in free-living human subjects and support the notion that the role of habitual NFD in cardiometabolic health is more complex than previously thought. Additional prospective cohort studies with multiple time points of diet and clinical assessments and appropriately powered randomized controlled trials are needed to disentangle the complex interplay between habitual NFD, circadian timing of food intake, and eating frequency in cardiometabolic health across the adult life course and to investigate their influence on other clinically relevant cardiovascular risk factors, including cardiac inflammatory markers. The potential underlying mechanistic pathways including circadian rhythms, the gut microbiome, hormonal factors such as cortisol, and cell signaling pathways related to metabolic homeostasis (e.g., mammalian target of rapamycin) [31,40] also warrant investigation. Moreover, time-stamped diet data from multi-ethnic cohorts of US adults are necessary to decipher potential racial and ethnic differences in these associations and to identify contributing environmental, psychosocial, and clinical factors. Elucidating these relationships will be necessary to inform more targeted interventions addressing eating patterns for lowering cardiometabolic risk.

## Figures and Tables

**Table 1:** Descriptive characteristics of the study population at baseline and follow-up ^a, b^.

Characteristics (Mean (SD)/% (*n*))	Baseline (n = 116)	One-Year (n = 99)	*p*-Value
Socio-Demographic Characteristics			
Age (y)	33 (12)	35 (13)	<0.001
Less than or Equivalent to College Education (%)	66.4% (77)	59.6% (59)	0.709
Health Insurance (%)	57.8% (67)	66.7% (66)	0.230
Racial/Ethnic Minority (%)	77.6% (90)	75.8% (75)	0.877
Hispanic Ethnicity (%)	44.8% (52)	47.5% (47)	0.802
Eating Pattern Characteristics			
Average Time of First Meal	8:32 (1:34)	9:16 (1:46)	0.001
Average Time of Last Meal	20:08 (1:45)	19:58 (1:20)	0.046
Average Nightly Fasting Duration (h)	12.4 (2.0)	13.3(2.3)	<0.001
Average Number of Eating Occasions	3.9 (1.1)	3.7 (1.0)	0.001
Cardiovascular Risk Factors			
AHA LS7	10.7 (2.0)	10.2 (2.6)	0.049
Smokers (%)	6.9% (8)	5.0% (5)	0.780
Moderate-to-Vigorous Intensity Physical Activity (h/day)	4.1 (5.3)	5.7 (7.5)	0.113
BMI (kg/m^2^)	25. 7 (5.4)	25.9 (5.3)	0.197
Overweight and Obesity (%)	48.3% (56)	51.5% (51)	0.736
WC (inches)	35.4 (4.9)	36.2 (4.7)	0.004
At-Risk WC (>35 inches)	44.8% (52)	43.4% (43)	0.946
SBP (mmHg)	116.0 (12.1)	115.2 (11.9)	0.459
DBP (mmHg)	72.7 (10.6)	72.2 (9.7)	0.542
Fasting glucose (mg/dL)	84.7 (21.2)	92.6 (15.8)	<0.001
HbA1c (%)	5.5 (0.7)	5.5 (1.0)	0.919
Total Cholesterol (mg/dL)	169.7 (32.5)	172.5 (29.8)	0.762
LDL (mg/dL)	96.6 (28.6)	95.8 (25.4)	0.270
HDL (mg/dL)	57.5 (11.8)	60.2 (12.9)	0.020
Triglycerides (mg/dL)	78.1 (36.2)	82.1 (42.6)	0.309

^a^ AHA: American Heart Association; BMI: body mass index; DBP: diastolic blood pressure; HbA1c: hemoglobin A1c (a glycated form of hemoglobin); LS7: Life’s Simple 7; SBP: systolic blood pressure; SD: standard deviation; LDL: low-density lipoprotein; HDL: high-density lipoprotein; WC: waist circumference. ^b^ T-tests and Chi-square tests were used to examine differences in participant characteristics at baseline vs. 1-y follow up.

**Table 2:** Linear Regression Models for Cross-sectional and Prospective Associations of Habitual Nightly Fasting Duration (NFD) with Cardiovascular Health and Cardiometabolic Risk Factors ^a^.

Per 1-h Increase in Average Nightly Fasting Duration						
	Cross-Sectional Analysis of Baseline Data (n = 116) ^b,c^		Cross-Sectional Analysis of 1-y Data (n = 99) ^b,c^		Prospective Associations of Baseline Exposures with Outcomes at 1-y (n = 99) ^b,c^	
	Β (SE)	*p*-Value	Β (SE)	*p*-Value	Β (SE)	*p*-Value
CVH (AHA LS7 score)	−0.09 (0.09)	0.329	−0.22 (0.09)	**0.016**	−0.22 (0.11)	**0.050**
BMI (kg/m^2^)	−0.02 (0.25)	0.941	0.33 (0.29)	0.256	0.31 (0.34)	0.357
WC (inches)	−0.22 (0.22)	0.323	0.09 (0.25)	0.721	0.20 (0.29)	0.501
SBP (mmHg)	0.85 (0.56)	0.130	1.00 (0.51)	0.055	0.47 (0.81)	0.566
DBP (mmHg)	0.44 (0.49)	0.378	1.08 (0.40)	0.009	1.74 (0.63)	0.007
Fasting glucose (mg/dl)	0.66 (1.05)	0.532	−0.26 (0.97)	0.791	1.17 (1.07)	0.279
HbA1c (%)	0.04 (0.03)	0.260	−0.001 (0.06)	0.983	0.05 (0.07)	0.436
Total Cholesterol (mg/dl)	−0.19 (1.43)	0.895	0.04 (1.56)	0.980	−2.90 (1.72)	0.097
HDL (mg/dl)	−0.14 (0.58)	0.807	−0.57 (0.76)	0.454	−0.62 (0.88)	0.482
LDL (mg/dl)	−0.50 (1.28)	0.696	0.75 (1.36)	0.584	−2.60 (1.51)	0.088
Triglycerides (mg/dl)	2.29 (1.77)	0.198	−0.84 (2.00)	0.677	1.61 (2.77)	0.562

^a^ CVH: cardiovascular health; AHA: American Heart Association; BMI: body mass index; DBP: diastolic blood pressure; HbA1c: glycated hemoglobin; LS7: Life’s Simple 7; SBP: systolic blood pressure; SE: standard error; WC: waist circumference; ^b^ Models were adjusted for age, race/ethnicity, health insurance, and physical activity. For AHA LS7 score, models were adjusted for age, race/ethnicity, and health insurance; ^c^ Additional adjustment for BMI slightly attenuated magnitude of NFD with DBP associations but not their statistical significance. Specifically, BMI-adjusted results were the following: cross-sectional associations of NFD with DBP at 1 y (β(SE) = 0.98 (0.40), *p* = 0.017); prospective associations of baseline NFD with DBP at 1 y (β(SE) = 1.59 (0.62), *p* = 0.011).

**Table 3:** Linear Regression Models for Cross-sectional and Prospective Associations of Average Timing of First Eating Occasion with Cardiovascular Health and Cardiometabolic Risk Factors ^a^.

Per 30-min Delay in Average Timing of First Eating Occasion						
	Cross-Sectional Analysis of Baseline Data (n = 116) ^b,c^		Cross-Sectional Analysis of 1-y Data (n = 99) ^b,c^		Prospective Associations of Baseline Exposures with Outcomes at 1-y (n = 99) ^b,c^	
	Β (SE)	*p*-Value	Β (SE)	*p*-Value	Β (SE)	*p*-Value
CVH (AHA LS7 score)	−0.07 (0.06)	0.234	−0.11 (0.06)	0.058	−0.20 (0.08)	0.013
BMI (kg/m^2^)	−0.04 (0.16)	0.793	0.12 (0.18)	0.498	0.30 (0.23)	0.184
WC (inches)	−0.12 (0.15)	0.415	0.11 (0.15)	0.482	0.30 (0.19)	0.123
SBP (mmHg)	0.37 (0.37)	0.313	0.58 (0.32)	0.074	0.42 (0.54)	0.439
DBP (mmHg)	0.46 (0.32)	0.151	0.53 (0.26)	0.042	1.18 (0.42)	0.006
Fasting glucose (mg/dl)	0.54 (0.69)	0.434	−0.01 (0.60)	0.990	1.43 (0.70)	0.045
HbA1c (%)	0.03 (0.02)	0.154	−0.002 (0.04)	0.954	0.05 (0.05)	0.281
Total Cholesterol (mg/dl)	−0.57 (0.94)	0.547	0.35 (0.97)	0.716	−1.77 (1.15)	0.128
HDL (mg/dl)	−0.29 (0.38)	0.450	−0.29 (0.47)	0.546	−0.30 (0.59)	0.615
LDL (mg/dl)	−0.75 (0.83)	0.370	0.59 (0.85)	0.490	−1.82 (1.00)	0.075
Triglycerides (mg/dl)	2.32 (1.15)	0.045	0.15 (1.25)	0.908	1.75 (1.84)	0.375

^a^ AHA: American Heart Association; BMI: body mass index; DBP: diastolic blood pressure; HbA1c: glycated hemoglobin; LS7: Life Simple 7; SBP: systolic blood pressure; SE: standard error; WC: waist circumference; ^b^ Models were adjusted for age, race/ethnicity, health insurance and physical activity. For AHA LS7 score, models were adjusted for age, race/ethnicity and health insurance; ^c^ Additional adjustment for BMI did not alter statistical significance of cross-sectional associations of timing of the first eating occasion with triglyceride levels at baseline (β(SE) = 2.35 (1.14), *p* = 0.042), but attenuated associations with DBP at 1 y (β(SE) = 0.49 (0.25), *p* = 0.056). Adjustment for BMI did not significantly change observed prospective associations of baseline timing of the first eating occasion with DBP (β(SE) = 1.05 (0.41), *p* = 0.013) and fasting glucose at 1 y (β(SE) = 1.45 (0.72), *p* = 0.046).

**Table 4:** Linear Regression Models for Cross-sectional and Prospective Associations of Habitual Eating Frequency with Cardiovascular Health and Cardiometabolic Risk Factors ^a^.

Per One Additional Eating Occasion Each Day						
	Cross-Sectional Analysis of Baseline Data (n = 116) ^b,c^		Cross-Sectional Analysis of One-Year Data (n = 99) ^b,c^		Prospective Associations of Baseline Exposures with Outcomes at 1-y (n = 99) ^b,c^	
	Β (SE)	*p*-Value	Β (SE)	*p*-Value	Β (SE)	*p*-Value
CVH (AHA LS7 score)	0.01 (0.16)	0.958	0.38 (0.21)	0.073	0.22 (0.19)	0.245
BMI (kg/m^2^)	−0.26 (0.46)	0.581	−1.06 (0.65)	0.109	−0.79 (0.53)	0.156
WC (inches)	−0.02 (0.42)	0.962	−0.58 (0.56)	0.302	−0.27 (0.46)	0.563
SBP (mmHg)	−0.95 (1.05)	0.368	−0.06 (1.20)	0.960	−0.84 (1.27)	0.509
DBP (mmHg)	−1.94 (0.90)	0.033	−1.25 (0.95)	0.193	−3.37 (0.96)	0.001
Fasting glucose (mg/dl)	−1.50 (1.94)	0.442	−1.17 (2.19)	0.596	−0.86 (1.68)	0.611
HbA1c (%)	−0.06 (0.06)	0.349	−0.05 (0.14)	0.715	−0.09 (0.10)	0.374
Total Cholesterol (mg/dl)	0.15 (0.17)	0.372	0.49 (3.53)	0.889	4.45 (2.69)	0.102
HDL (mg/dl)	0.26 (1.07)	0.806	0.56 (1.73)	0.747	0.40 (1.38)	0.772
LDL (mg/dl)	1.53 (2.37)	0.521	−0.32 (3.10)	0.918	3.75 (2.36)	0.116
Triglycerides (mg/dl)	0.58 (3.31)	0.862	1.42 (4.55)	0.756	1.55 (4.33)	0.721

^a^ AHA: American Heart Association; BMI: body mass index; DBP: diastolic blood pressure; HbA1c: glycated hemoglobin; LS7: Life Simple 7; SBP: systolic blood pressure; SE: standard error; WC: waist circumference; ^b^ Models were adjusted for age, race/ethnicity, health insurance, and physical activity. For AHA LS7 score, models were adjusted for age, race/ethnicity and health insurance; ^c^ Additional adjustment for BMI did not alter significance of cross-sectional associations of eating frequency with DBP at baseline (β(SE) = −1.78 (0.86), *p* = 0.040) or prospective associations of baseline eating frequency with DBP at 1 y (β(SE) = −3.04 (0.95), *p* = 0.002).
